# The Structure and the Outcome of Telephone-Based Cardiac Consultations During Lockdown: A Lesson From COVID-19

**DOI:** 10.7759/cureus.11585

**Published:** 2020-11-20

**Authors:** Osama M Alhadramy

**Affiliations:** 1 Internal Medicine, College of Medicine, Taibah University, Medina, SAU

**Keywords:** telephone, cardiac, consultation, lockdown, covid-19

## Abstract

Background

In response to COVID-19, Saudi Arabia as many countries, implemented “lockdown” to contain the epidemic. This resulted in suspension of all outpatient services. The reliability of the alternative telecommunication cardiac services during that time is not well studied. Accordingly, the objective of this study is to describe the structure of the telephone-based cardiac consultation (TBCC) and to explore its outcome.

Methods

This is a cross-sectional study that has a prospective follow up on patients who underwent medical intervention. During the period of lockdown, Alre’aiah health care society in Almadinah Almunawwarah, Saudi Arabia, provided a community health service. This was achieved by announcing telephone numbers for consultations in most specialties. This study includes all TBCC of a single cardiologist. Detailed demographic data, medical, social and drug histories of the patients were collected in charts. Individuals were requested to measure blood pressure (BP) and heart rate (HR) at the time of TBCC. Accordingly, cardiovascular assessment and appropriate intervention were executed. Patients who needed medical intervention were followed up in one week. The data were analyzed using appropriate statistical methods.

Results

From 01 April till 15 June 2020, a total of 168 individuals sought TBCC. Their median age was 51.5 ± 12.7 years, and (57.1%) were females. Healthy individuals constituted (33.9%), and (59.9%) were non-smokers. The most common reported medical illnesses were hypertension (27.3%), diabetes (23.8%), heart failure (16.1%), and coronary artery disease (14.9%). Palpitations were encountered by 58 patients. None of them had high-risk features or cardiac disease. Stress, excessive smoking, and caffeine intake were thought to be responsible for palpitations in 52 individuals who were reassured and educated, and newly diagnosed hypertension was established prospectively in eight patients and they were started on medications. Chest pain was reported by 51 individuals. The diagnosis of typical angina was made in nine patients and they were instructed to seek emergency care. Atypical angina pain was established in 10 cases who were advised to seek formal consultation once lockdown ends. Reassurance was achieved in 32 individuals who had features of non-angina pain. Uncontrolled hypertension was reported by 32 patients. Blood pressure control was achieved prospectively in 70% of these patients who followed up by adjusting their antihypertensive drugs. Twenty-seven patients with heart failure complained of worsening shortness of breath. New York Heart Association (NYHA) class 1-2 was reported by 21 patients, and they were managed by doubling diuretic dose, 19 of them followed back and reported significant improvement. NYHA class 3-4 was established in 6 patients and they were instructed to seek emergency care.

Conclusions

When standard face-to-face cardiac consultations are compromised, a structured TBCC is considered feasible, seems effective, and promising alternative method of delivering the utmost cardiac care to the community. When conducted properly, it is useful to triage patients.

## Introduction

In December 2019, a series of patients with a severe form of lower respiratory tract infection in Wuhan, China were reported [1]. Shortly, the coronavirus disease 2019 (COVID-19) was prescribed and became a pandemic. As of April 28, 2020, the disease spread in 185 countries which resulted in more than three million reported cases, and a mortality that exceeded 200 thousand casualties [2].

To prevent the spread of this pandemic, many countries have declared ‘lockdown’, infection control policies, social distancing, and self-isolation which restricted the movement of people [3]. Primary healthcare during that time was critically compromised because healthcare sectors were already overwhelmed by services delivered to COVID-19 patients, and the spread of the infection among health care providers [4]. Therefore, the health care systems had to be restructured to minimize individuals contact with healthcare providers, inhibit or reschedule hospital visits and outpatient clinics, and postpone any elective procedures. As an alternative option there has been a growing interest in providing telehealth, also called telemedicine, which provides personal distancing precautions with patient care. Telecommunication (TC) options such as telephone or video consultations have played a key role during that difficult time in the diagnosis and the management of many diseases, particularly in those with cardiovascular diseases [5]. Such services include review of patient medical history, current complaints, drug history, functional status and subsequently a diagnosis can be established, and management plan can be executed [6]. In Saudi Arabia, the ministry of health offered smartphone applications, which enabled the public to obtain medical advice via phone calls [7].

Because patients with underlying cardiovascular diseases are at greater risk for severe COVID-19 complications, more extreme home isolation measures were implemented for them, particularly patients with severe heart failure or valvular heart disease [8]. This has led to a significant reduction in outpatient clinics, procedures, hospitalizations, cases with myocardial infarction, coronary angiogram, cardiac interventions and open heart surgery [9,10]. The role of telemedicine was well studied, but little is known about the accuracy and the reliability of the other forms of TC cardiac services. Accordingly, the objectives of this study are to describe the structure of telephone-based cardiac consultation (TBCC) on individuals who were unable to obtain standard face-to-face cardiac care, and to explore its outcome.

## Materials and methods

This study was conducted from 01 April to 15 June 2020. It included all consecutive patients aged >18-year-old who sought cardiology consultations by calling the telephone number that was assigned to a single cardiologist. This remote community service was an initiative of Alre’aiah health care society in Almadinah Almunawwarah region, Saudi Arabia, throughout the period of lockdown. Alre’aiah is a growing non-profit health care organization that was found in 2001 in Almedinah Almunawwarah city, Saudi Arabia, which delivers a wide spectrum of medical services to the community, and has a solid charitable program which served more than 8000 individuals in 2019. It is licensed by the Ministry of Health, and under the jurisdiction of the Ministry of Human Resources and Social Development. Because of the impact and the long period of lockdown, which limited the population from seeking health care, the medical board in the organization decided to create an initiative of remote clinical services to the community that covers many specialties like Internal Medicine and its specialties, General Surgery, Pediatrics, Psychiatry and Obstetrics and Gynecology. Each subspecialty is covered by more than one consultant, and each had a designated telephone number, and they were available on specific days and times. Consultants who voluntarily participated in this initiative were from different health care institutes in Almadinah Almunawwarah city. A schedule was made which included names of the consultants, their specialties and availability time, and was publically released in social media. Consultant received telephone calls from individuals with regard specific concerns, and subsequently plans were executed and, then prospectively followed up. In this study, TBCCs were scheduled every Saturday from 4 to 10 pm. Charts were made that included demographic data, medical, social, drug histories and the reason for consultation. All individuals were requested to record BP and HR by digital devices during telephone call. After recording blood pressure and heart rate cardiac assessment was made. Subsequently, plans were executed which included reassurance or medical intervention. Patients who underwent medical intervention were prospectively requested to call back at one-week interval to evaluate outcomes. Data were collected and analyzed using appropriate statistical methods.

## Results

During the lockdown period, a total of 168 individuals sought TBCC. The mean age of the cohort was 51.5 ± 12.7 years, and (57.1%) were females. Healthy individuals constituted (33.9%), the most common underlying medical illnesses were hypertension (27.3%), diabetes (23.8%), heart failure (16.1%), and (14.9%) were known to have coronary artery disease. The majority of them were Saudis (66.7%), and (59.9%) were non-smoker. The summary of their characteristics are listed in Table 1.

**Table 1:** Selected baseline characteristics

Characteristics	N= 168 (%)
Age in years, mean ± SD	51.5 ± 12.7
Male	73 (42.9)
Female	95 (57.1)
Saudi nationality	112 (66.7)
Non-Saudi nationality	56 (33.3)
Non-smoker	94 (55.9)
Smoker	74 (44.1)
Health	57 (33.9)
Hypertension	46 (27.3)
Diabetes	40 (23.8)
Heart Failure	27 (16.1)
Coronary artery disease	25 (14.9)

Reason for consultations

Palpitations, which were described as feeling of fast or bouncing heart beats, were reported by 58 cases, their mean age was 40 ± 12.7 years. These symptoms were encountered by 32 females and 26 males. This complaint was experienced by 49 smokers and 9 non-smokers. The majority of the individuals were healthy (75.9%), and only 14 patients were known to have hypertension.

Chest pain was reported by 51 individuals. Their mean age was 44 ± 6.1 years, and the majority of them were females (62.7%). This symptom was encountered by 25 patients with coronary artery disease, and by 26 healthy individuals.

Thirty-two individuals complained of uncontrolled hypertension. All of these patients were known to have pre-existing hypertension on medications. The mean age of these patients was 47 ± 8.2 years. It was reported by 20 males and 12 females.

Worsening shortness of breath was reported by 27 patients, their mean age was 57 ± 6.1 years, and (59.3%) of them were males. All of these patients were known to have heart failure on medications. The number of patients with ejection fraction (EF) < 40% was 18, and the rest of the patients did not know the exact function.

**Figure 1:**
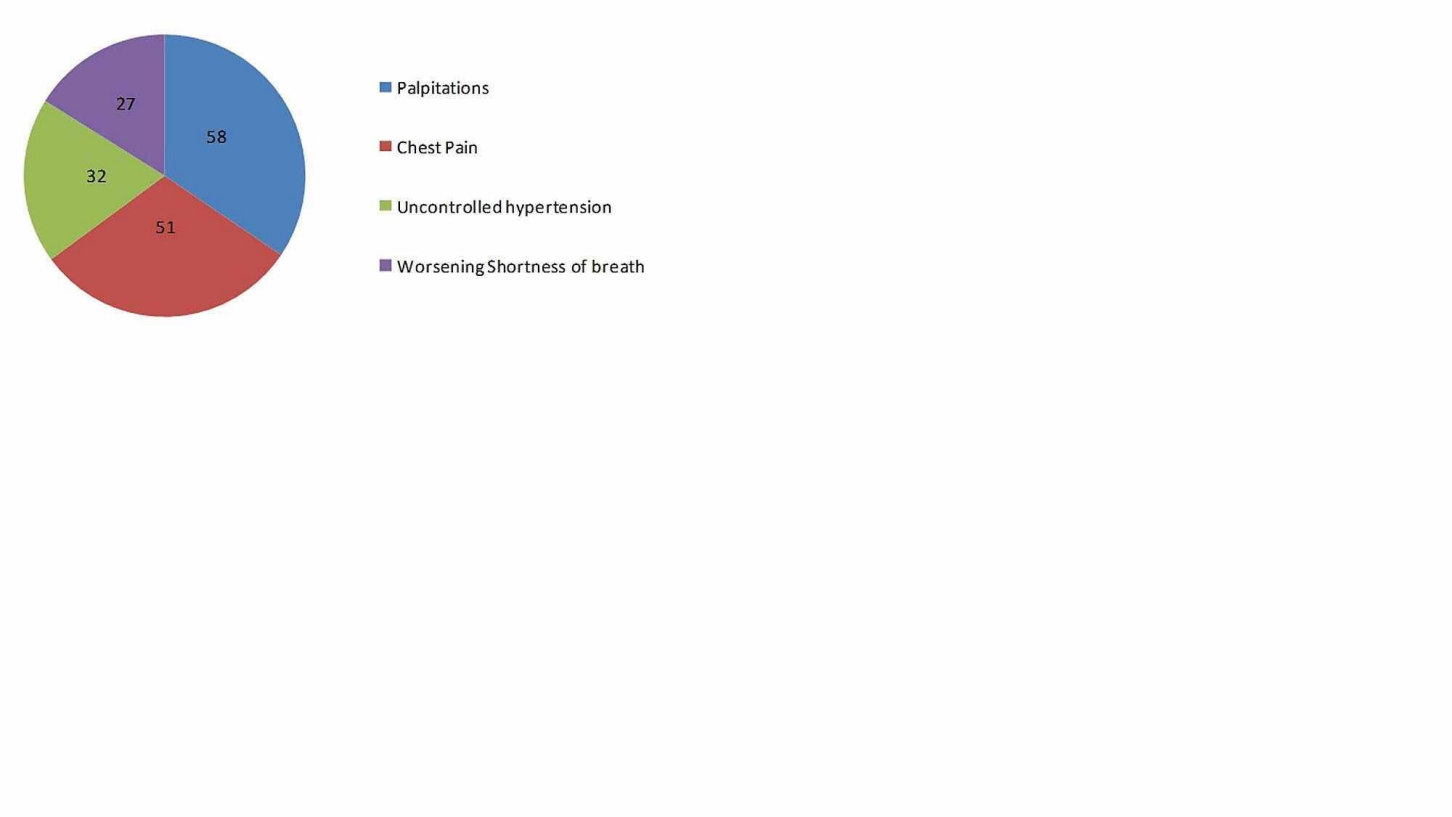
Reasons for telephone consultations (n = 168)

The reasons for telephone consultations are shown in Figure 1 and selected baseline characteristics of these patients are listed in Table 2.

**Table 2:** Distribution of selected baseline characteristics according to the reason of the consultation

Reason for consultation	Characteristics	Number (%)
Palpitations (n=58)	Age in years, mean ± SD	40 ± 12.7
	Male	26 (44.8)
	Female	32 (55.2)
	Healthy individuals	44 (75.8)
	Smokers	49 (84.5)
	Hypertension	14 (24.2)
	Non-smokers	9 (15.5)
Chest pain (n=51)	Age in years, mean ± SD	44 ± 6.1
	Male	11 (37.3)
	Female	40 (62.7)
	Coronary artery disease	25 (49)
	Healthy individuals	26 (51)
Uncontrolled hypertension	Age in years, mean ±	47 ± 8.2
	Male	20 (62.5)
	Female	12 (37.5)
	Hypertension	32 (100)
Worsening shortness of breath (n=27)	Age in years, mean ± SD	57 ± 6.1
	Male	16 (59.3)
	Female	11 (40.7)
	Ejection fraction < 40%	18 (66.7)
	Ejection fraction not known	9 (33.3)

Outcome

Palpitations

None of the 58 cases who experienced palpitation had syncope, family history of sudden cardiac death or exertional symptoms. These attacks were not sudden nor associated with sweating. In 52 patients, the BP and HR measurements during TBCC were within the normal range. In these cases, palpitations were linked to increased nicotine consumption and caffeine intake, and also lack of sleep. Reassurance was achieved in these 52 individuals and they were advised to decrease caffeine intake and quit or decrease cigarette smoking and to seek emergency care if they experience worsening symptoms.

**Figure 2:**
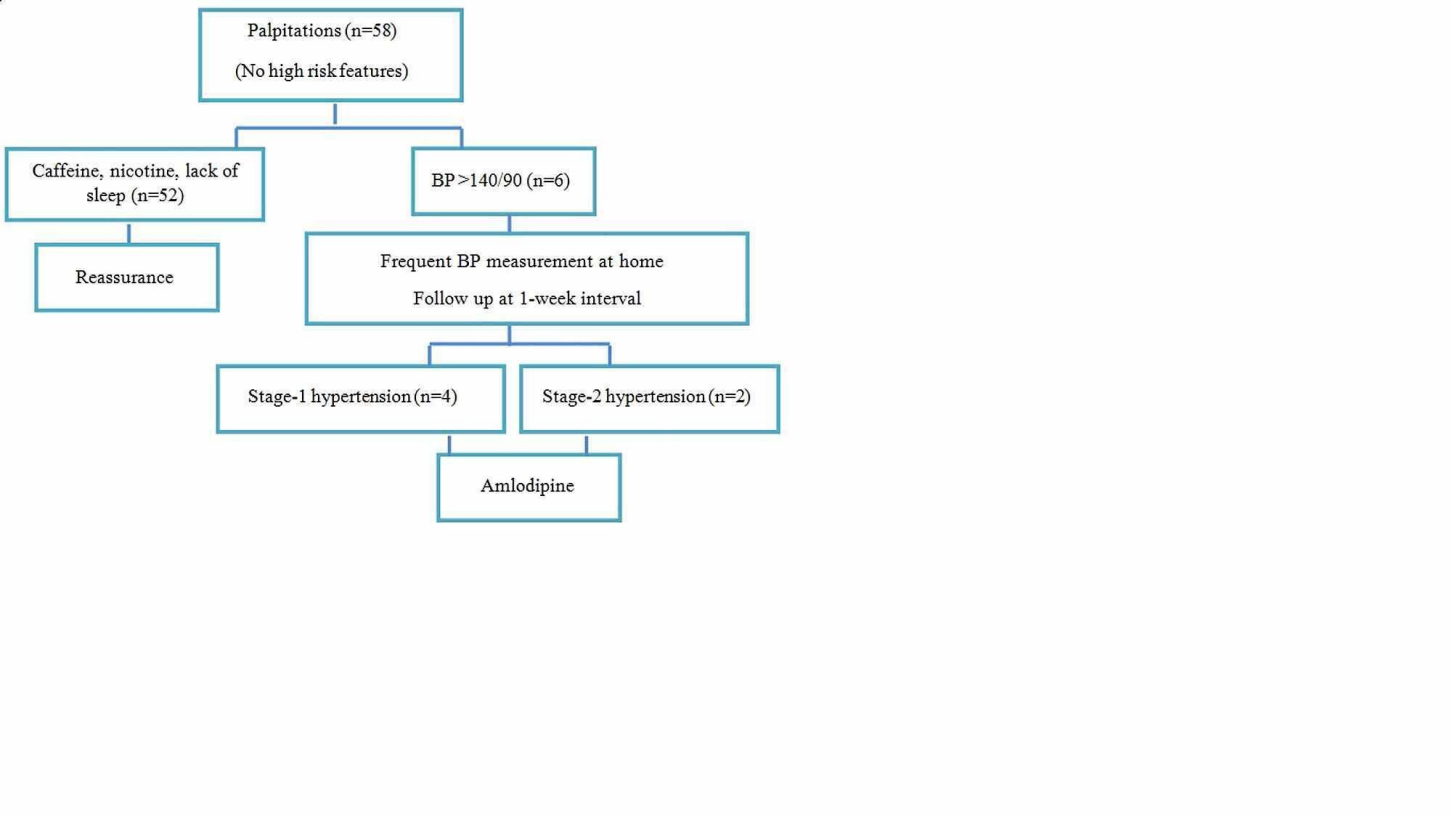
Outcome of patients with palpitations

In six individuals, the measurements of BP during the consultation were >140/90 mmHg. These patients were instructed to measure BP in both arms at rest four times daily for one week, and they were prospectively followed up at one-week interval. BP measurements were reviewed and subsequently four patients were found to have stage-1 hypertension and two patients were within stage-2 hypertension. Amldoipine as anti-hypertensive medicine was used because of its safety profile. These outcomes are shown in Figure 2.

Chest pain

**Figure 3:**
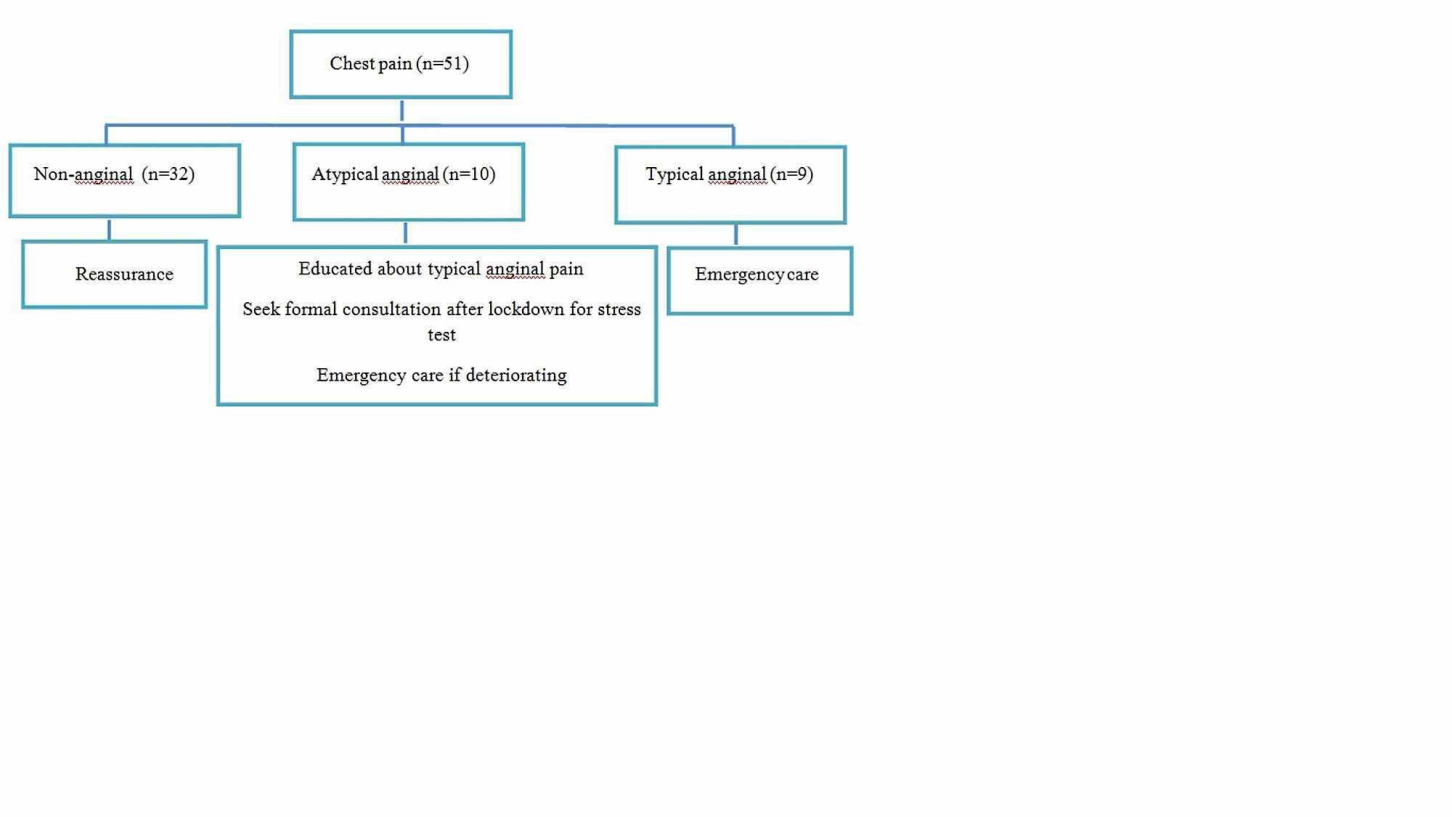
Outcome of patients with chest pain

After detailed analyses of the pain in 51 individuals, the diagnosis of non-angina pain was established in 32 individuals. They were reassured, educated about the typical angina pain, and were advised to seek emergency care if they experience worsening symptoms. Atypical angina pain was identified in 10 cases and they were educated about the typical angina pain and were encouraged to seek formal evaluation once lockdown ends. The diagnosis of typical angina pain was made in nine patients and they were instructed to seek emergency care. These outcomes are shown in Figure 3.

Uncontrolled hypertension

**Figure 4:**
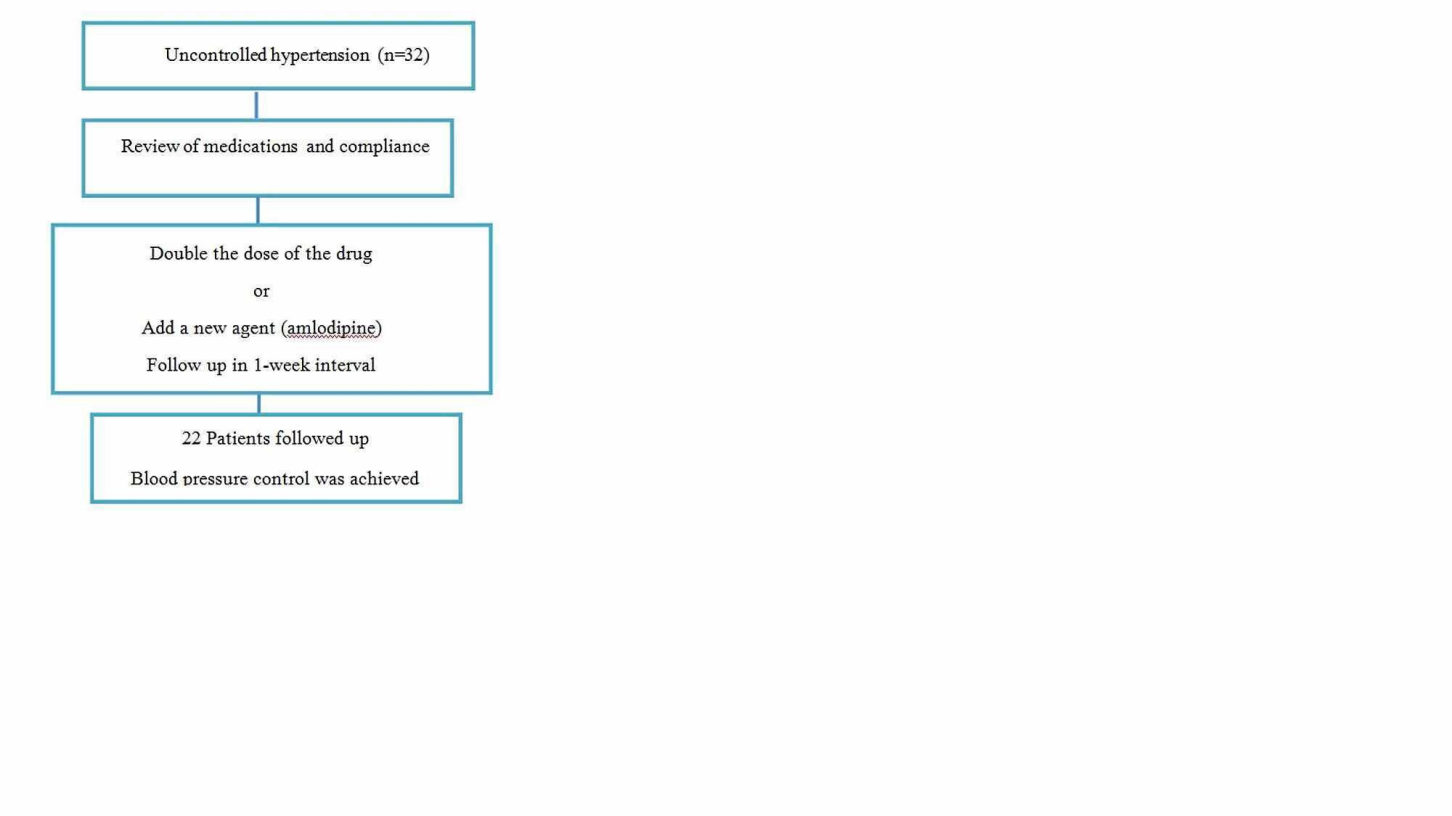
Outcome of patients with hypertension

Thirty-two patients reported uncontrolled hypertension despite being compliant with medications. After reviewing anti-hypertensive agents, the dose of the drugs were doubled or another agent was introduced. They were instructed to call the ambulance if BP becomes uncontrolled. Prospective follow-up was made in 22 individuals who reported satisfactory control. These outcomes are shown in Figure 4.

Worsening shortness of breath

**Figure 5:**
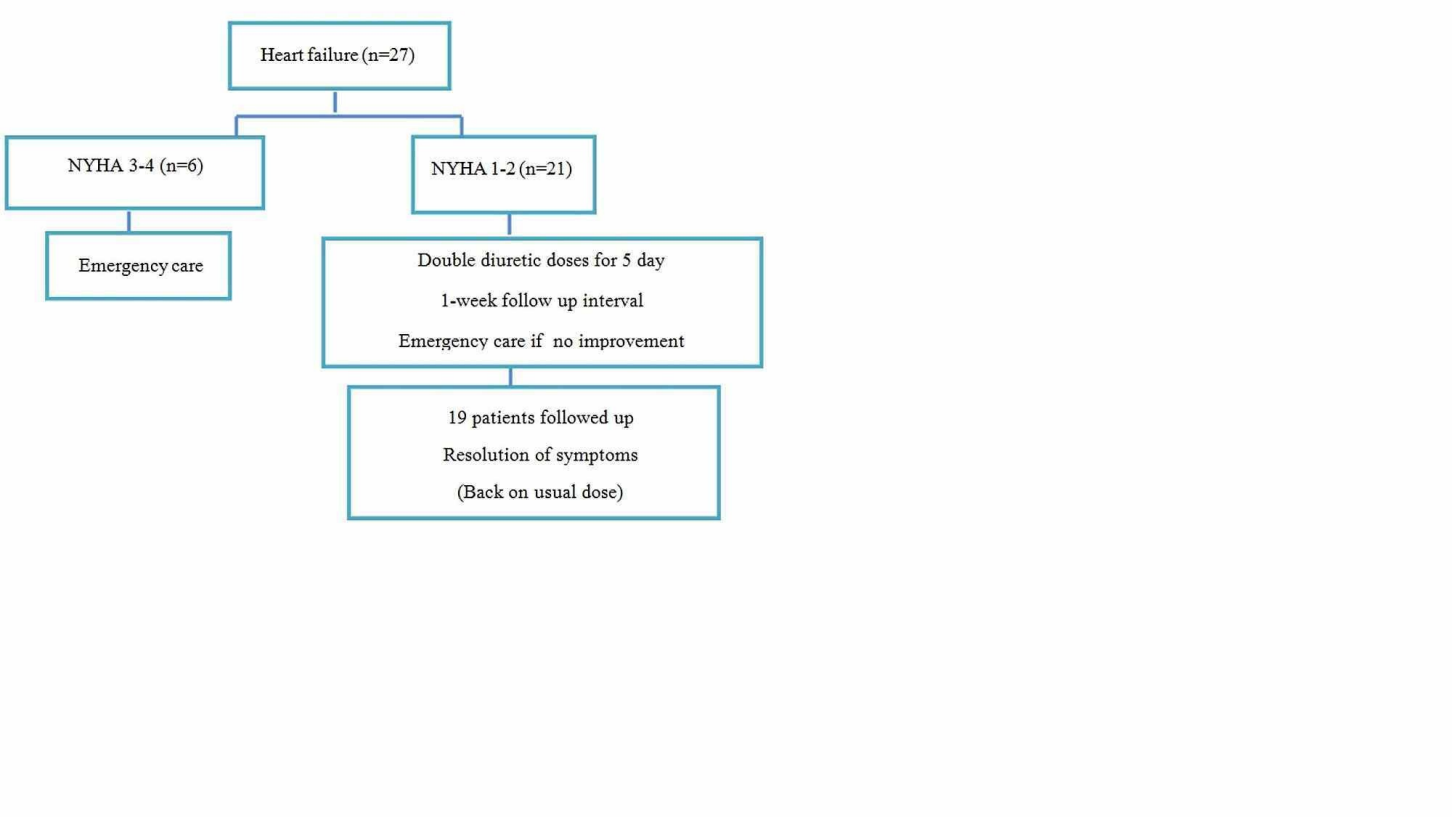
Outcome of patients with worsening shortness of breath NYHA: New York Heart Association class of shortness of breath

Twenty-seven patients reported worsening shortness of breath despite being compliant with medical therapy. NYHA class 1-2 was identified in 21 patients. The medications of these patients were reviewed and accordingly were instructed to double the dose of the diuretics for five days and then resume the usual daily dose. They were instructed to seek emergency care if symptoms worsen. One-week prospective follow-up on 19 patients revealed full recovery and subsequently they resumed the usual dose. NYHA class 3-4 was identified in six individuals and they were advised to seek emergency care. These outcomes are shown in Figure 5.

## Discussion

This is a cross-sectional study that has a prospective follow up on patients who underwent medical intervention. A total of 168 individuals sough TBCC during the period of lockdown. Females constituted (57.1%) of the cohort, and the mean age of the cohort was 51.5 ± 12.7 years. Thirty-three percent of these individuals were healthy and the majority were Saudis and non-smokers. The most common past medical history in this cohort was hypertension, diabetes, coronary artery disease and heart failure, respectively.

During the pandemic, a cross-sectional study that looked at emergency room visits in 24 hospitals in the United States found >40% reduction rate from January to April 2020 [11]. Similar observations were consistent in Italy, Austria and Spain [12-14]. This could be attributed to three reasons, the fear of infection, some hospitals were dedicated only to COVID-19 patients, and finally the delay in making policies and procedures toward the care of non-COVID individuals. For these reasons, healthcare systems have transformed tinto virtual- and telecommunication-care [15].

To the best of my knowledge, this is the first study in Saudi Arabia and the Middle East that describes the most common cardiovascular illnesses encountered by the community during the long lockdown period, and sheds light on the outcome of structured TBCC. People around the world have never experienced a similar situation. Accordingly, studies that explore the common cardiovascular-related illnesses in any period of the curfew are critical. Such studies can help authorities to prioritize health care plans, and to establish an effective and alternative option for remote health care delivery to the community should any similar disaster happen in the future. This is critical because the whole world is awaiting the second wave of COVID-19 in the winter.

Palpitations, which were described as fast or bouncing beats, were the leading reason for consultation. None of these individuals experienced high-risk symptoms, and the majority were free from any medical illnesses. This group of individuals was relatively younger than the overall cohort and the majority were females. Assurance was achieved in (89.7%) of them in whom these symptoms were attributed to increased caffeine and tobacco consumption, and also lack of sleep. This is likely in association with the mental and psychological impact of the epidemic. This is similar to what is known in the literature. In a large-scale cross-sectional study the epidemic-related insomnia, anxiety and depression were seen in (30%) of the cases [16]. It was also estimated that 40% of the smokers experienced an increase in smoking frequency during the period of lockdown [17]. This is consistent with the findings of this cohort.

A careful history is reliable in distinguishing cardiac and non-cardiac etiology of palpitation. It has been suggested that patients with panic attacks were more likely to describe palpitations as "racing" or "bouncing” beats, and this pattern was not associated with significant arrhythmia on ECG or 24-h ambulatory monitor [18]. Also, there is a good correlation between cardiac arrhythmia as cause of palpitations and the presence of structural heart disease, syncope, family history of sudden cardiac death, or the presence of cardiac symptoms [19]. None of these high-risk features were identified in the history of this cohort.

The majority of the individuals who reported palpitations were females. This is likely related to their higher stress level compared to males during the lockdown. This was also shown in some studies [20].

Newly diagnosed hypertension was established in 6 patients who encountered palpitation. In the initial TBCC, these patients were having BP >140/90mmHg. They were instructed to self-measure and record BP at home four times per day at rest for seven days. These instructions were adapted from the 2019 Nice Guidelines of hypertension [21]. Follow up was achieved in all of them, and their BP measurements met definitions of stage-1 or -2 hypertension. After careful counselling and education, medical therapy was established with amlodipine as calcium channel blocker because of its safety profile. They were instructed to seek formal consultation once the lockdown ends. Also, there were 32 patients who reported uncontrolled hypertension despite being compliant with medications. It is known that the long period of lockdown was associated with worsening glycemic and blood pressure control [22]. This is likely because of the stress level, dietary habits and weight gain. Hypertension guidelines recommend self-measurement of BP at home in the diagnosis, follow up, and also for the initiation or titration of antihypertensive medications [23]. After obtaining BP measurements and detailed medical and drug histories, appropriate therapeutic strategies were executed by either doubling the dose or starting a new anti-hypertensive agent. Seventy percent of these patients were followed up and reported satisfactory control. This telephone-based intervention has been described before and was found to be an effective method to improve compliance in patients with uncontrolled or newly diagnosed hypertension [24]. The RITE-BP trial also showed that blood pressure control was successfully achieved in patients through telephone calls and for 6 months follow up [25].

Chest pain was encountered by 51 individuals. The majority of them were females, and 25 patients were known to have coronary artery disease. The diagnosis of anginal pain was made in 9 patients, and they were advised to seek emergency care. Atypical angina pain was established in 10 individuals whom were educated about the typical angina pain, and were also encouraged to seek formal consultation after lockdown for further evaluation. Finally, non-anginal pain was made in 32 individuals and they were reassured. This simple classification of the chest pain, that depends on history is established in the guidelines [26]. It is based on the 3 criteria: retrosternal chest that is exacerbated by exertion and relieved by rest or nitroglycerine. The presence of 3 criteria defines typical angina pain, 2 criteria makes the pain atypical, and the presence of 1 criterion is diagnostic for non-anginal pain. Patients with atypical angina pain should undergo further evaluation electively for further stratification [27]. For that reason, telephone-triage of patients with chest pain by physicians has been shown to be safe, effective and decreased the mortality [28].

Symptoms of heart failure were found in 27 patients, EF<40% was known by 18 individuals. NYHA class 1-2 were encountered by 77% and they were managed by doubling the diuretic dose for five days with instruction to follow-up in one week. Twenty-four patients followed back and resumed their usual dose. There were six cases with NYHA class 3-4 and were instructed to see emergency care. This remote management of heart failure patients has been validated in the literature and strongly advocated in the guidelines [29]. Telephone-based interventions in patients with chronic heart failure are well documented before the era of COVID-19 and has been shown to reduce mortality [30].

There are important messages in this study. Cardiovascular-related illnesses were common during the period of lockdown. Curfew significantly prevented individuals with- or without underlying cardiac diseases from seeking cardiac care. There were few options available to the general community to help them to achieve effective cardiac care. In Saudi Arabia, the Ministry of Health has launched smartphone applications and hot-line to allow the public to seek medical advice. However, these options may not be as effective as the structured TBCC because they are not conducted by specialized consultants. Telephone-based cardiac intervention has strong evidence in the literature and was well-validated before the area of COVID-19 when delivered properly. 

This study shows for the first time in the region that such services can be provided effectively and seem safe. Structured TBCC achieved better outcomes regarding medical interventions and also was efficient to triage sick individuals which might lead to better utilization of resources in case of a national crisis. Similar initiatives can play a critical role should another pandemic compromise the health care sectors.

Although there was proper documentation of data and follow up was achieved in most of the patients who underwent medical intervention, this study has multiple limitations. These consultations were only telephone-based and do not meet the specifications of telemedicine nor virtual clinics in which there is remote face-to-face encounter together with the availability of the investigations of the patients. However, there were few patients in this study who sent appropriate investigations like electrocardiograms or cardiac enzymes through WhatsApp, and accordingly, received better counselling. But this was difficult to apply to all individuals because most laboratories and other facilities were shutdown. Another important limitation is that all of the clinical data which were obtained over the telephone were subjective and they could have been under- or overestimated by the patients. Finally, the outcome of the patients who were advised to seek emergency care is not known but this was not part of the structure of the consultation.

## Conclusions

A structured TBCC is a promising effective and reliable way of delivering cardiac care to the community when there is compromise of the standard services. When delivered systematically, it can provide the general population the fundamental cardiac care. This form of telecommunication health services are particularly valuable to patients with cardiovascular diseases because it grants them a remote and efficient care.
